# Exploring the association between magnetocardiographic parameters and echocardiographic indices of concentric left ventricular remodeling in hypertension: a cross-sectional correlation study

**DOI:** 10.1016/j.clinsp.2026.101044

**Published:** 2026-07-29

**Authors:** Junyi Zhang, Jianfeng Zhang, Yang Duan, Yafeng Zhou

**Affiliations:** Department of Cardiology, The Fourth Affiliated Hospital of Soochow University, Medical Center of Soochow University, Suzhou Dushu Lake Hospital, China

**Keywords:** Magnetocardiography, Left ventricular remodeling, Hypertension, Echocardiography, Electrical remodeling, Electromechanical coupling

## Abstract

**Background:**

Left Ventricular Remodeling (LVR) in hypertension involves both structural and electrophysiological alterations. Magnetocardiography (MCG) non-invasively measures cardiac magnetic fields, potentially reflecting these changes. This study aimed to explore the association between MCG parameters and established Echocardiographic (ECHO) indices of concentric left ventricular remodeling in hypertensive patients.

**Methods:**

In this cross-sectional study, 220 hypertensive patients underwent both ECHO and 9-channel MCG. Patients were categorized based on ECHO into Non-LVR (n = 105) and concentric remodeling groups (n = 115). Fifteen MCG parameters derived from the QRS complex and R-wave were analyzed. Correlations between MCG and ECHO parameters (Left Ventricular Mass Index [LVMI], Interventricular Septal thickness [IVSd], Left Ventricular Posterior Wall thickness [LVPWd], Relative Wall Thickness [RWT]) were assessed. A multivariate associative model combining selected MCG parameters was developed in a 70% subset (n = 154), and its association with ECHO-defined concentric structural phenotype was tested in a separate 30% validation subset (n = 66).

**Results:**

After rigorous correction for multiple testing, several MCG parameters demonstrated statistically significant correlations with specific ECHO indices. The maximum current moment of the QRS complex (QRS_MCM) showed positive correlations with IVSd (*r* = 0.388, p < 0.00083), LVPWd (*r* = 0.333, p < 0.00083), and RWT (*r* = 0.392, p < 0.00083). The relative timing parameters QRS_CA ratio and QRS_FMA ratio exhibited significant positive correlations with both IVSd and RWT (*r* = 0.299-0.339, all p < 0.00083). Patients in the ECHO-defined concentric remodeling group exhibited a distinct MCG profile characterized by increased current moments, altered current angles, and higher depolarization timing ratios compared to the Non-LVR group. The multivariate associative model, incorporating QRS_MCM, R_CA, QRS_CA ratio, and QRS_FMA ratio, showed a strong association with concentric structural phenotype in the development set (AUC = 0.887, 95% CI 0.832–0.942). Critically, this association remained robust and significant when the model was applied to the independent validation set (AUC = 0.856, 95% CI 0.763–0.948). The internal validation confirmed the stability of this associative pattern, though the model is not intended for clinical diagnostic use.

**Conclusion:**

Specific magnetocardiographic parameters, particularly those reflecting the global strength of ventricular depolarization (QRS_MCM) and the relative timing of electrical events within the cardiac cycle, are significantly and robustly associated with echocardiographic indices of concentric left ventricular remodeling in hypertension. These cross-sectional findings establish a measurable link between MCG-based electrophysiological features and concentric structural alterations in hypertensive patients. Further prospective studies are needed to explore the temporal relationship between electrical and structural remodeling and to determine whether MCG offers insights beyond those obtainable from simpler, more widely available tools such as the ECG.

## Highlights

• Magnetocardiography (MCG) parameters correlate with echocardiographic left ventricular remodeling in hypertension.
• QRS Maximum Current Moment (QRS_MCM) is associated with wall thickness and relative wall thickness.
• Relative timing ratios (QRS_CA ratio, QRS_FMA ratio) are linked to concentric geometry.
• A four-parameter MCG model shows stable association with concentric structural phenotype.

## Introduction

Systemic arterial hypertension remains a paramount global public health challenge, exerting its deleterious effects primarily on the heart, brain, and kidneys. The heart, as a central target organ, undergoes a spectrum of adaptive and maladaptive changes in response to chronic pressure overload, collectively termed hypertensive heart disease. A cornerstone of this pathological continuum is Left Ventricular Remodeling (LVR), a complex process involving alterations in myocardial mass, chamber geometry, tissue composition, and cellular electrophysiology.^1^^,^^2^ LVR is not a uniform entity but manifests in distinct patterns, with concentric remodeling ‒ characterized by increased relative wall thickness without an increase in mass ‒ and concentric hypertrophy ‒ involving both increased wall thickness and mass ‒ being hallmarks of pressure overload states like hypertension.^3^^,^^4^

The clinical significance of detecting LVR early cannot be overstated. It represents a critical intermediate phenotype that signifies target organ damage and is a powerful, independent predictor of adverse cardiovascular outcomes, including heart failure, atrial fibrillation, ventricular arrhythmias, and sudden cardiac death.^5^^,^^6^ The transition from asymptomatic hypertension to symptomatic heart failure is often mediated by the progression of LVR. Consequently, accurate identification and characterization of LVR are essential for refined risk stratification and for guiding timely therapeutic interventions aimed at reversing or attenuating remodeling, a concept central to modern cardiology.^7^^,^^8^

In clinical practice, two-dimensional transthoracic Echocardiography (ECHO) serves as the primary tool and indispensable tool for the non-invasive assessment of LVR. It provides direct, high-resolution imaging of cardiac structures, allowing for the precise measurement of wall thicknesses, chamber dimensions, and the calculation of derived indices such as Left Ventricular Mass Indexed to body surface area (LVMI) and Relative Wall Thickness (RWT).^9^^,^^10^ These metrics form the basis of widely accepted classification schemes that categorize ventricular geometry into normal, concentric remodeling, concentric hypertrophy, and eccentric hypertrophy. Despite its ubiquity and utility, ECHO is fundamentally a tool for evaluating cardiac structure and function. It provides limited direct insight into the concurrent electrophysiological remodeling that is increasingly recognized as an integral component of the hypertensive heart phenotype.

Electrophysiological alterations, including changes in action potential duration, calcium handling, and connexin expression, are known to occur in hypertrophied and failing myocardium and may even precede detectable structural changes.^11^^,^^12^ The standard 12-lead Electrocardiogram (ECG) has historically been used to infer structural changes through voltage criteria for left ventricular hypertrophy (e.g., Sokolow-Lyon index). However, these criteria suffer from well-documented limitations, including poor sensitivity (often below 50%) and susceptibility to confounding factors such as body habitus, lung disease, and pericardial effusion.^13^^,^^14^ The ECG is largely insensitive to the early, concentric remodeling phase, where mass is not yet increased. Thus, there is interest in exploring whether electrophysiology-based tools can complement structural imaging.

Magnetocardiography (MCG) emerges as a compelling candidate to address this gap. MCG measures the extremely weak magnetic fields (on the order of picotesla) produced by the same ionic currents that generate the body surface potentials recorded by ECG. However, as a contactless technique, the magnetic signal is less distorted by the varying electrical conductivity of intervening tissues (lungs, fat, bone), providing a potentially more direct and localized view of the underlying cardiac electrical source.^15^^,^^16^ Furthermore, advanced MCG systems with multi-channel sensor arrays allow for the construction of magnetic field maps over the chest, from which parameters beyond simple waveform amplitude can be derived, such as the current moment vector, its orientation (angle), and the spatial distribution of magnetic fields.^17^ These features suggest that MCG may offer advantages in detecting subtle electrophysiological disturbances associated with early myocardial remodeling that are not apparent on standard ECG.

Previous investigations into the utility of MCG for conditions involving ventricular hypertrophy or hypertension have been conducted, but are marked by heterogeneity and inconclusive findings. Studies have often employed parameters borrowed from ischemic heart disease research or focused on repolarization (T-wave) abnormalities, with inconsistent results regarding their correlation with hypertrophy.^18^^,^^19^ A systematic exploration of MCG parameters specifically in relation to the detailed, quantitative structural indices provided by ECHO ‒ particularly differentiating between concentric remodeling and hypertrophy ‒ is lacking. Moreover, many prior studies have been limited by small sample sizes and a lack of internal validation for any proposed parameter combinations.

Therefore, this study was conceived as an exploratory, descriptive correlation study with the following specific aims:

1. To systematically investigate associations between a wide array of MCG parameters ‒ spanning amplitude, timing, angular, and spatial domains ‒ and key quantitative ECHO indices of concentric left ventricular remodeling (LVMI, IVSd, LVPWd, RWT) in a well-characterized cohort of hypertensive patients.
2. To compare MCG parameter profiles between patients with and without ECHO-defined concentric structural phenotypes.
3. To explore, in an internally validated manner, whether a combination of MCG parameters exhibits a stable and robust associative pattern with the concentric structural phenotype defined by ECHO.

This work is deliberately positioned not as a diagnostic accuracy study, but as a detailed correlation and association study. Its purpose is to map the landscape of relationships between MCG signals and ECHO-defined structural changes, thereby identifying the most promising MCG markers associated with hypertensive heart disease. It remains unclear whether MCG offers incremental value over standard, widely available tools such as the 12-lead ECG. This study does not aim to establish clinical utility but rather to systematically characterize associations between MCG parameters and echocardiographic indices of left ventricular remodeling.

## Methods

### Study design and participants

This investigation was designed as a single-center, observational, cross-sectional study. The primary objective was to explore associations between parameters derived from two distinct modalities ‒ MCG and ECHO ‒ within the same patient population. A total of 220 consecutive patients who were admitted to the Department of Cardiology for evaluation and management of hypertension between October 2021 and August 2023 were prospectively enrolled. This cross-sectional study adheres to the Strengthening the Reporting of Observational Studies in Epidemiology (STROBE) statement guidelines for reporting observational studies. The study protocol received full approval from the Institutional Review Board/Ethics Committee of the Fourth Affiliated Hospital of Soochow University. The study was conducted in strict accordance with the ethical principles outlined in the Declaration of Helsinki. Prior to any study-related procedures, written informed consent was obtained from every participant.

The diagnosis of hypertension was established based on the criteria set forth in the 2018 Chinese Guidelines for the Prevention and Treatment of Hypertension.^20^ This included: 1) A Systolic Blood Pressure (SBP) ≥ 140 mmHg and/or Diastolic Blood Pressure (DBP) ≥ 90 mmHg measured in the clinical setting on at least three separate occasions in patients not taking antihypertensive medications, or 2) A documented history of hypertension with current use of antihypertensive medication. All enrolled patients were aged 18-years or older.

To ensure a relatively homogeneous cohort focused on hypertensive remodeling and to minimize confounding from other significant cardiac pathologies, a comprehensive set of exclusion criteria was applied. Patients were excluded if they had: constrictive pericarditis or significant pericardial effusion; moderate or severe native valvular heart disease (stenosis or regurgitation) involving the mitral or aortic valves; a history of myocardial infarction, percutaneous coronary intervention with stent implantation within the past 6 months, or known severe coronary artery stenosis (> 70% in a major epicardial vessel); diagnosed cardiomyopathies (dilated, restrictive, hypertrophic, or alcoholic); symptomatic heart failure classified as New York Heart Association (NYHA) functional class III or IV; frequent ventricular arrhythmias (e.g., non-sustained ventricular tachycardia, frequent premature ventricular complexes); implanted cardiac devices (pacemakers, defibrillators); metallic implants or dental work that could cause significant signal artifact during MCG; severe hepatic or renal dysfunction (defined by laboratory criteria); current pregnancy; or incomplete clinical, echocardiographic, or MCG data necessary for the analysis.

### Echocardiographic protocol and group definition

All participants underwent a comprehensive transthoracic echocardiographic examination performed by experienced sonographers using a commercially available ultrasound system (Philips EPIQ CVx) equipped with a phased-array transducer. Examinations were performed according to contemporary guideline recommendations.^21^ Patients were examined in the left lateral decubitus position, and standard parasternal and apical views were acquired and stored for offline analysis.

Key measurements were performed on 2D images averaged over three consecutive cardiac cycles:

Left Ventricular Internal Diameter at End-diastole (LVEDd) and End-Systole (LVESd) were measured from the parasternal long-axis view using the leading-edge method.

Interventricular Septal thickness at end-diastole (IVSd) and Left Ventricular Posterior Wall thickness at end-diastole (LVPWd) were also measured at end-diastole from the same view. Left Ventricular Mass (LVM) was calculated using the validated Devereux formula: $LVM\;\left(g\right)=0.8\times \left\{1.04\times [{\left(IVSd+LVEDd+LVPWd\right)}^{3}-{\left(LVEDd\right)}^{3}]\right\}\;+\;0.6$.^22^ To account for differences in body size, LVM was indexed to body surface area (BSA, calculated using the DuBois formula) to obtain the Left Ventricular Mass Index (LVMI, g/m^2^). Relative Wall Thickness (RWT), an index of concentricity, was calculated as: RWT=(2×LVPWd)/LVEDd. Based on established criteria integrating LVMI and RWT,^9^^,^^20^ patients were classified into the following groups:

Normal Geometry (Non-LVR group): Defined by normal LVMI (men < 115 g/m^2^, women < 95 g/m^2^) and RWT ≤ 0.42.

Concentric Remodeling (CR): Defined by normal LVMI and RWT > 0.42.

Concentric Hypertrophy (CH): Defined by increased LVMI (men ≥ 115 g/m^2^, women ≥ 95 g/m^2^) and RWT > 0.42.

For the primary group-wise analyses, patients meeting criteria for CR or CH were pooled to form the concentric structural phenotype group (hereafter referred to as the concentric remodeling/hypertrophy group). A detailed comparison across all three subgroups is provided in Supplementary Table S3.

### Magnetocardiography acquisition and parameter derivation

MCG recordings were performed using a nine-channel low-temperature superconducting magnetocardiography system (CARDIOMOX MCG-9, Oxford Cardimerk Medical Instruments Co., Ltd.) in a magnetically shielded room. Patients were positioned supine, and a simultaneous lead II ECG was recorded for timing reference. The sensor array moved to 36 predefined measurement points in a 6×6 grid. Raw data were digitized, filtered (0.1–100 Hz), and averaged using R-wave triggering.

Fifteen parameters were derived, including:

QRS_Integral: Time integral of magnetic field magnitude over QRS duration.
QRS_MCM, R_MCM: Maximum current moment during QRS and at R-wave peak.
QRS_CA, R_CA: Current angle at maximum QRS and at R-wave peak.
QRS_FMA, R_FMA: Field mapping angle.
QRS_PD, R_PD: Polar distance.
R_CA_TIME, QRS_CA_TIME, QRS_FMA_TIME: Absolute times from QRS onset.
R_CA ratio, QRS_CA ratio, QRS_FMA ratio: Relative timing ratios (absolute time / RR interval.

### Statistical analysis

Data analysis was performed using IBM SPSS Statistics (Version 26.0) and R (Version 4.2.2). Continuous variables were compared using *t*-test, ANOVA, Mann-Whitney *U*, or Kruskal-Wallis tests as appropriate. Categorical variables were compared using the chi-square or Fisher’s exact test.

Correlation Analysis: Pearson or Spearman correlation coefficients were calculated for each of the 15 MCG parameters with each of the 4 ECHO parameters (60 tests). The Bonferroni correction was applied, setting significance at α = 0.05 / 60 = 0.00083. A sensitivity analysis using the Benjamini-Hochberg False Discovery Rate (FDR) procedure was also performed and is presented in Supplementary Table S2.

Group Comparisons: MCG parameters were compared between the Non-LVR and concentric remodeling/hypertrophy groups. Subgroup comparisons across Non-LVR, CR, and CH are presented in Supplementary Table S3.

Multivariate Associative Model: The cohort was randomly divided into a development set (70%, n = 154) and a validation set (30%, n = 66) using stratified sampling. Candidate MCG parameters (p < 0.10 in univariate logistic regression) were entered into a multivariable binary logistic regression model with backward stepwise selection. The final model was applied to the validation set with fixed coefficients to assess stability. Model performance was evaluated using Area Under the ROC curve (AUC), sensitivity, specificity, Positive Predictive Value (PPV), and Negative Predictive Value (NPV). Calibration was assessed in the development set, and limitations due to sample size in the validation set are acknowledged.

For all tests except the pre-specified correlation analysis, a two-tailed p-value < 0.05 was considered statistically significant.

## Results

### Baseline demographic, clinical, and echocardiographic characteristics

The study cohort comprised 220 hypertensive patients with an overall median age of 64 years (IQR: 54–71). Age did not differ significantly between groups (concentric remodeling/hypertrophy group: 60 [50.5–71] vs. Non-LVR: 67 [55–71], p = 0.122). The sample included 113 males (51.4%) and 107 females (48.6%).

As shown in Table 1, the concentric remodeling/hypertrophy group had a higher body surface area (1.87±0.19 vs. 1.80±0.20 m^2^, p = 0.006). The group showed higher values for most structural ECHO parameters, with statistically significant differences for IVSd (11.2±1.6 vs. 9.5±1.4 mm, p < 0.001), LVPWd (10.9±1.5 vs. 9.4±1.2 mm, p < 0.001), LVMI (112.3 [99.5, 128.0] vs. 81.4 [72.1, 91.9] g/m^2^, p < 0.001), and RWT (0.46±0.06 vs. 0.38±0.04, p < 0.001). The difference in LVM was of borderline significance (p = 0.048). There were no significant differences in resting heart rate or blood pressure between groups. Of the 115 patients in the concentric remodeling/hypertrophy group, 65 (56.5%) were classified as Concentric Remodeling (CR) and 50 (43.5%) as Concentric Hypertrophy (CH).

**Table 1:** Comparison of basic characteristics, ECHO and MCG parameters between hypertensive left ventricular remodeling group and concentric remodeling group.

**Indicator**	**Left ventricular remodeling (n = 115)**	**Concentric remodeling (n = 105)**	**p**
Age (yrs)	60 (50.5∼71)	67 (55∼71)	0.122
Height (cm)	163 (157.5∼169)	163 (158∼168)	0.242
Weight (kg)	66 (58∼75)	66 (60∼74)	0.932
LVM	176 (142.42∼220)	165 (133∼194.44)	0.048
BSA	1.87 (1.68∼2.06)	1.8 (1.60∼2.00)	0.006
LVMI	80.4 (62.36∼98.6)	75.38 (58.34∼84.04)	0.015
IVSd (cm)	1.1 (0.98∼1.2)	0.9 (0.81∼0.95)	0.001
LVPWd (cm)	1 (0.9∼1.1)	0.87 (0.80∼0.94)	0.001
RWT	0.44 (0.43∼0.46)	0.35 (0.33∼0.37)	0.001
QRS_integral	2394.57 (1369.8∼4392.84)	1304.57 (396.09∼2040.07)	0.001
QRS_MCM	269.4 (168.8∼352.6)	152.9 (119∼176.9)	0.001
QRS_CA	164.9 (127.6∼179.15)	172.4 (154.5∼177.6)	0.278
QRS_FMA	153.43 (94.73∼165.96)	108.43 (63.43∼180)	0.186
QRS_PD	9.2 (8.5∼10)	8.5 (8∼10.2)	0.075
R_MCM	243.8 (148.3∼342.5)	143.6 (114.3∼166.3)	0.001
R_CA	23.6 (2.7∼43.15)	49.8 (31.1∼81.2)	0.001
R_FMA	51.34 (45∼69.885)	51.34 (45∼57.41)	0.44
R_PD	6.4 (5.8∼7.2)	6.4 (5.8∼6.7)	0.75
R_CA_TIME (ms)	282 (248∼318)	296 (260∼332)	0.316
QRS_CA_TIME (ms)	299 (271.5∼354)	326 (260∼357)	0.52
QRS_FMA_TIME (ms)	324 (295∼354)	330 (298∼363)	0.535
R_CA_TIME ratio	0.35 (0.31∼0.37)	0.31 (0.29∼0.33)	0.001
QRS_CA_TIME ratio	0.38 (0.36∼0.42)	0.35 (0.32∼0.37)	0.001
QRS_FMA_TIME ratio	0.4 (0.38∼0.42)	0.35 (0.32∼0.0.3)	0.001

MCG, Magnetocardiography; LVM, Left Ventricular Mass; BSA, Body Surface Area; LVMI, Left Ventricular Mass Index; IVSd, Interventricular Septum Dimension; LVPWd, Left Ventricular Posterior Wall dimension; RWT, Relative Wall Thickness; MCM, Maximum Current Moment; CA, Current Angle; FMA, Field Map Angle; PD, Pole Distance.

### Association between MCG and ECHO parameters

After applying the Bonferroni-corrected significance threshold (p < 0.00083), a focused set of statistically significant associations emerged, as summarized in Table 2.

**Table 2:** Correlation analysis between ECHO parameters and MCG parameters.

**Indicator**	**LVMI**		**LVSd**		**LVPWd**		**RWT**	
	***r* (95%CI)**	**p**	***r* (95%CI)**	**p**	***r* (95%CI)**	**p**	***r* (95%CI)**	**p**
QRS_Integral	0.061 (-0.072, 0.192)	0.368	0.092 (-0.041, 0.222)	0.172	0.079 (-0.054, 0.209)	0.242	0.164 (0.032, 0.291)	0.015
QRS_MCM	0.197 (0.066, 0.322)	0.003	0.388 (0.267, 0.497)	0.001	0.333 (0.208, 0.447)	0.001	0.392 (0.271, 0.500)	0.001
R_MCM	0.212 (0.081, 0.366)	0.002	0.323 (0.198, 0.438)	0.001	0.313 (0.187, 0.429)	0.001	0.301 (0.174, 0.418)	0.001
R_CA	-0.181 (-0.307, -0.049)	0.007	-0.206 (-0.330, -0.075)	0.002	-0.249 (-0.370, -0.120)	0.001	-0.207 (-0.331, -0.076)	0.002
R_CA ratio	0.077 (-0.056, 0.207)	0.256	0.276 (0.148, 0.396)	0.001	0.187 (0.055, 0.313)	0.005	0.285 (0.158, 0.404)	0.001
QRS_CA ratio	0.134 (0.001, 0.262)	0.047	0.328 (0.203, 0.443)	0.001	0.262 (0.133, 0.383)	0.001	0.339 (0.215, 0.453)	0.001
QRS_FMA ratio	0.113 (-0.020, 0.242)	0.094	0.299 (0.172, 0.417)	0.001	0.281 (0.153, 0.401)	0.001	0.327 (0.202, 0.442)	0.001
QRS_CA	-0.199 (-0.324, -0.068)	0.003	-0.149 (-0.277, -0.016)	0.027	-0.069 (-0.200, 0.064)	0.306	-0.005 (-0.137, 0.127)	0.94
QRS_FMA	0.069 (-0.064, 0.199)	0.307	0.089 (-0.044, 0.219)	0.187	0.125 (-0.008, 0.253)	0.063	0.057 (-0.076, 0.188)	0.401
QRS_PD	0.057 (-0.076, 0.188)	0.397	0.039 (-0.094, 0.170)	0.568	0.066 (-0.067, 0.197)	0.333	0.015 (-0.117, 0.147)	0.83
R_FMA	0.087 (-0.046, 0.217)	0.197	0.096 (-0.037, 0.226)	0.156	0.168 (0.036, 0.295)	0.013	0.089 (-0.044, 0.219)	0.19
R_PD	0.244 (0.114, 0.366)	0.001	0.215 (0.084, 0.339)	0.001	0.172 (0.040, 0.298)	0.011	0.07 (-0.063, 0.200)	0.3
R_CA_TIME	0.069 (-0.062,0.203)	0.31	0.04 (-0.093, 0.171)	0.552	0.002 (-0.130, 0.134)	0.974	-0.089 (-0.219, 0.044)	0.188
QRS_CA_TIME	0.134 (0.001, 0.262)	0.047	0.123 (-0.010, 0.251)	0.069	0.101 (-0.032, 0.230)	0.136	-0.023 (-0.155, 0.109)	0.739
QRS_FMA_TIME	0.095 (-0.038, 0.225)	0.16	0.041 (-0.092, 0.172)	0.542	0.059 (-0.074, 0.190)	0.382	-0.072 (-0.202, 0.061)	0.289

CI, Confidence Interval; MCM, Maximum Current Moment; CA, Current Angle; FMA, Field Map Angle; PD, Pole Distance.

QRS_MCM demonstrated positive correlations with IVSd (*r* = 0.388, p < 0.0001), LVPWd (*r* = 0.333, p < 0.0001), and RWT (*r* = 0.392, p < 0.0001). Its correlation with LVMI did not survive correction (*r* = 0.197, p = 0.003). The relative timing parameters QRS_CA ratio and QRS_FMA ratio showed significant positive correlations with IVSd (*r* = 0.328 and *r* = 0.299, respectively) and RWT (*r* = 0.339 and *r* = 0.327, respectively), all p < 0.00083. R_PD showed a positive correlation with LVMI (*r* = 0.244, p = 0.0002). A sensitivity analysis using the Benjamini-Hochberg FDR procedure yielded consistent findings (Supplementary Table S2).

### Comparison of MCG parameters: non-LVR vs. concentric remodeling groups

**Figure 1:**
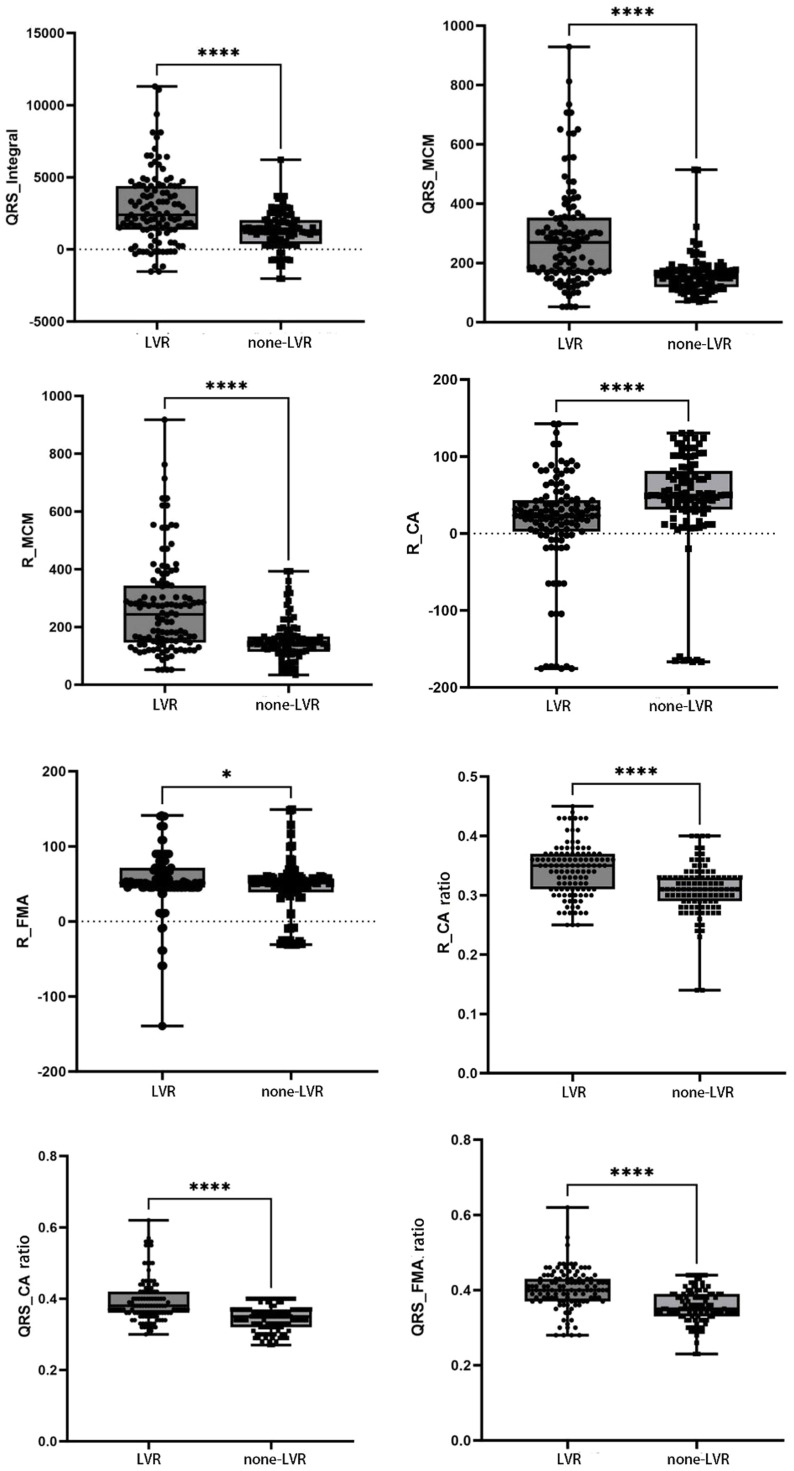
Comparison of Selected MCG Parameters Between Non-LVR and Concentric Remodeling Groups. Box plots showing the distribution of QRS_Integral, QRS_MCM, R_MCM, R_CA, R_FMA, R_CA ratio, QRS_CA ratio, and QRS_FMA ratio. ‘*’ denotes p < 0.05; ‘****’ denotes p < 0.001.

Comparison between the Non-LVR and concentric remodeling/hypertrophy groups revealed significant differences in several parameters (Fig. 1). QRS_MCM (224.7±58.1 vs. 185.4 ± 45.2 nAm, p < 0.001) and R_MCM (201.8 ± 49.1 vs. 160.8 ± 38.7 nAm, p < 0.001) were significantly higher in the concentric remodeling/hypertrophy group. R_CA was significantly smaller (39.6 ± 9.0 vs. 44.2 ± 8.9 degrees, p < 0.001). All three relative timing ratios were significantly higher: R_CA ratio (0.35 ± 0.05 vs. 0.31 ± 0.05, p < 0.001), QRS_CA ratio (0.39 ± 0.08 vs. 0.32 ± 0.06, p < 0.001), and QRS_FMA ratio (0.42 ± 0.09 vs. 0.34 ± 0.07, p < 0.001). QRS_Integral was also higher in the concentric remodeling/hypertrophy group (3274 ± 1092 vs. 2801 ± 892 pT × ms, p < 0.001).

### Comparison with standard ECG criteria

The authors also evaluated standard ECG voltage criteria in the same cohort. As shown in Table 3, the Sokolow-Lyon index, Cornell voltage, and Cornell product each showed modest discrimination for the concentric structural phenotype, with AUCs ranging from 0.62 to 0.68, sensitivities between 24% and 36%, and specificities of 86%–89%. By comparison, the four-parameter MCG associative model achieved an AUC of 0.856 in the validation set. However, this comparison is limited by the fact that the ECG analysis was performed in the same cohort used to develop the MCG model, and the two modalities differ substantially in cost, accessibility, and technical complexity. No direct head-to-head comparison with an independent reference standard was performed.

**Table 3:** Comparison of standard ECG voltage criteria and the four-parameter MCG associative model for discriminating the concentric structural phenotype.

**Criterion**	**AUC (95% CI)**	**Sensitivity (%)**	**Specificity (%)**	**PPV (%)**	**NPV (%)**
Sokolow-Lyon Index	0.62 (0.55–0.69)	24.3	89.5	‒	‒
Cornell Voltage	0.65 (0.58–0.72)	30.4	87.6	‒	‒
Cornell Product	0.68 (0.61–0.75)	35.7	86.2	‒	‒
MCG Four-Parameter Model (Validation Set)	0.856 (0.763–0.948)	100	57.8	70.2	100

AUC, Area Under the receiver operating Characteristic curve; PPV, Positive Predictive Value; NPV, Negative Predictive Value.

For ECG criteria, PPV and NPV are not reported due to the exploratory nature of the comparison and the absence of an independent reference standard.

Caution: Positive Predictive Value (PPV) and Negative Predictive Value (NPV) are dependent on the prevalence of concentric remodeling in this study cohort and may not generalize to other populations.

### Exploratory multivariate associative model

Univariate logistic regression identified eight MCG parameters with p < 0.10 (Table 4 in the original; now summarized). In the development set (n = 154), backward stepwise logistic regression retained four parameters as independently associated with the concentric structural phenotype: QRS_MCM, R_CA, QRS_CA ratio, and QRS_FMA ratio (Table 4).

**Table 4:** Univariate binary Logistic regression analysis of MCG parameters and left ventricular remodeling identified by echocardiography in hypertension.

**Indicator**	**Univariate binary Logistic regression**		
	**OR**	**95%CI**	**p**
QRS_Integral	1.0004	1.0002‒1.001	0.001
QRS_MCM	1.012	1.008‒1.016	0.001
R_MCM	1.01	1.006‒1.014	0.001
R_CA	0.993	0.988‒0.998	0.003
R_FMA	1.008	1.001‒1.016	0.029
R_CA ratio	19.18	12.24‒28.27	0.001
QRS_CA ratio	23.91	17.43‒33.65	0.001
QRS_FMA ratio	20.06	13.58‒30.19	0.001

OR, Odds Ratio; CI, Confidence Interval; MCM, Maximum Current Moment; CA, Current Angle; FMA, Field Map Angle.

**Figure 2:**
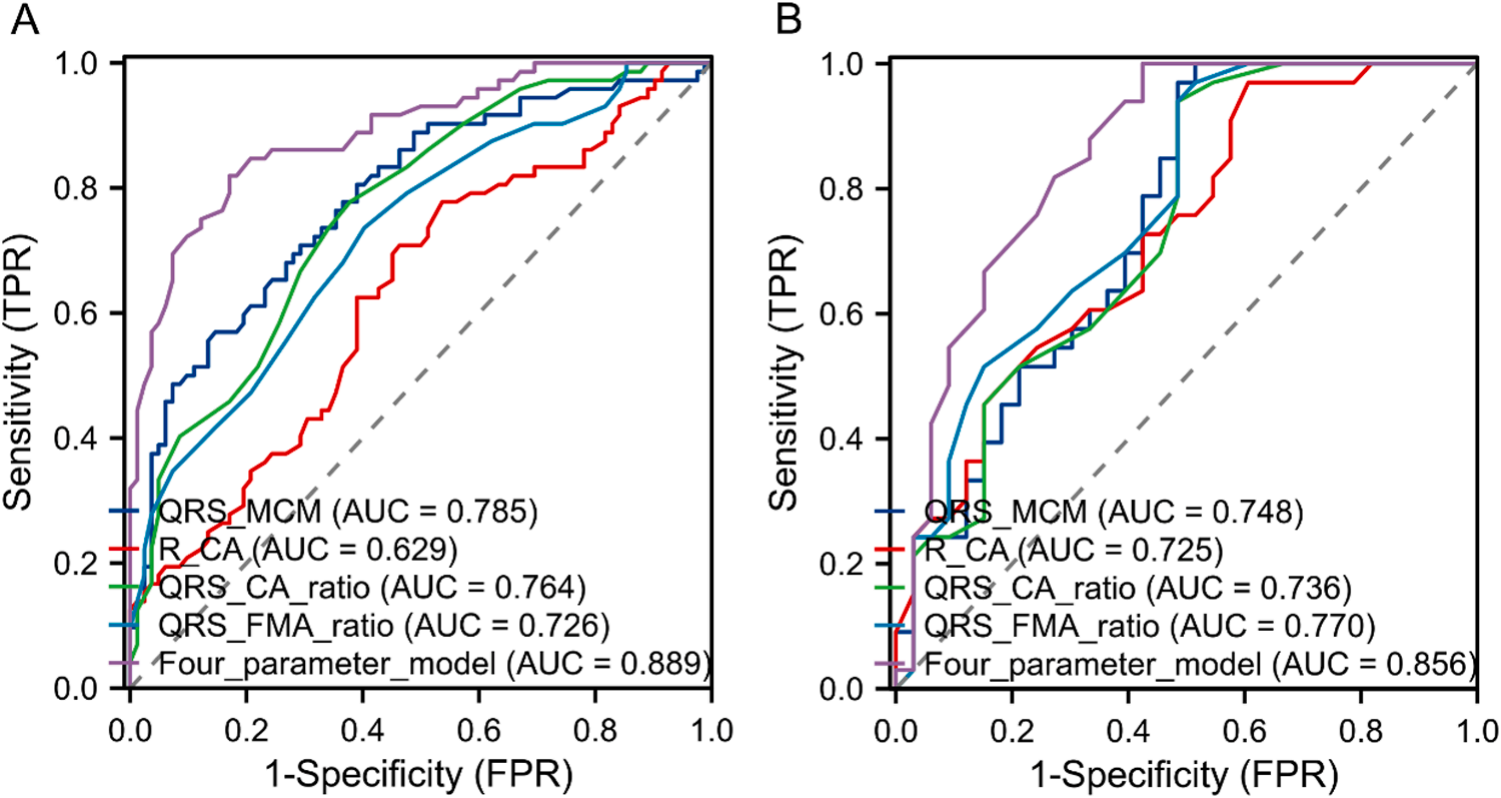
Receiver Operating Characteristic (ROC) Curves for the Four-Parameter MCG Associative Model. (A) ROC curves in the development set. (B) ROC curves in the validation set.

The model demonstrated a strong association with the ECHO-defined phenotype in the development set, with an AUC of 0.887 (95% CI 0.832–0.942; Fig. 2A). At the optimal cut-point (Youden index), sensitivity was 82.9%, specificity 82.5%, PPV 80.8%, and NPV 84.0%.

When applied to the independent validation set (n = 66) with fixed coefficients, the model maintained an AUC of 0.856 (95% CI 0.763–0.948; Fig. 2B). Notably, at the same probability threshold, sensitivity increased to 100% while specificity decreased to 57.8% (PPV 70.2%, NPV 100%). This shift reflects expected variability when a model optimized in one sample is evaluated in another, particularly with moderate sample sizes. The stability of the AUC suggests that the overall associative pattern is preserved, though the model’s probability estimates are not sufficiently calibrated for clinical use.

**Figure 3:**
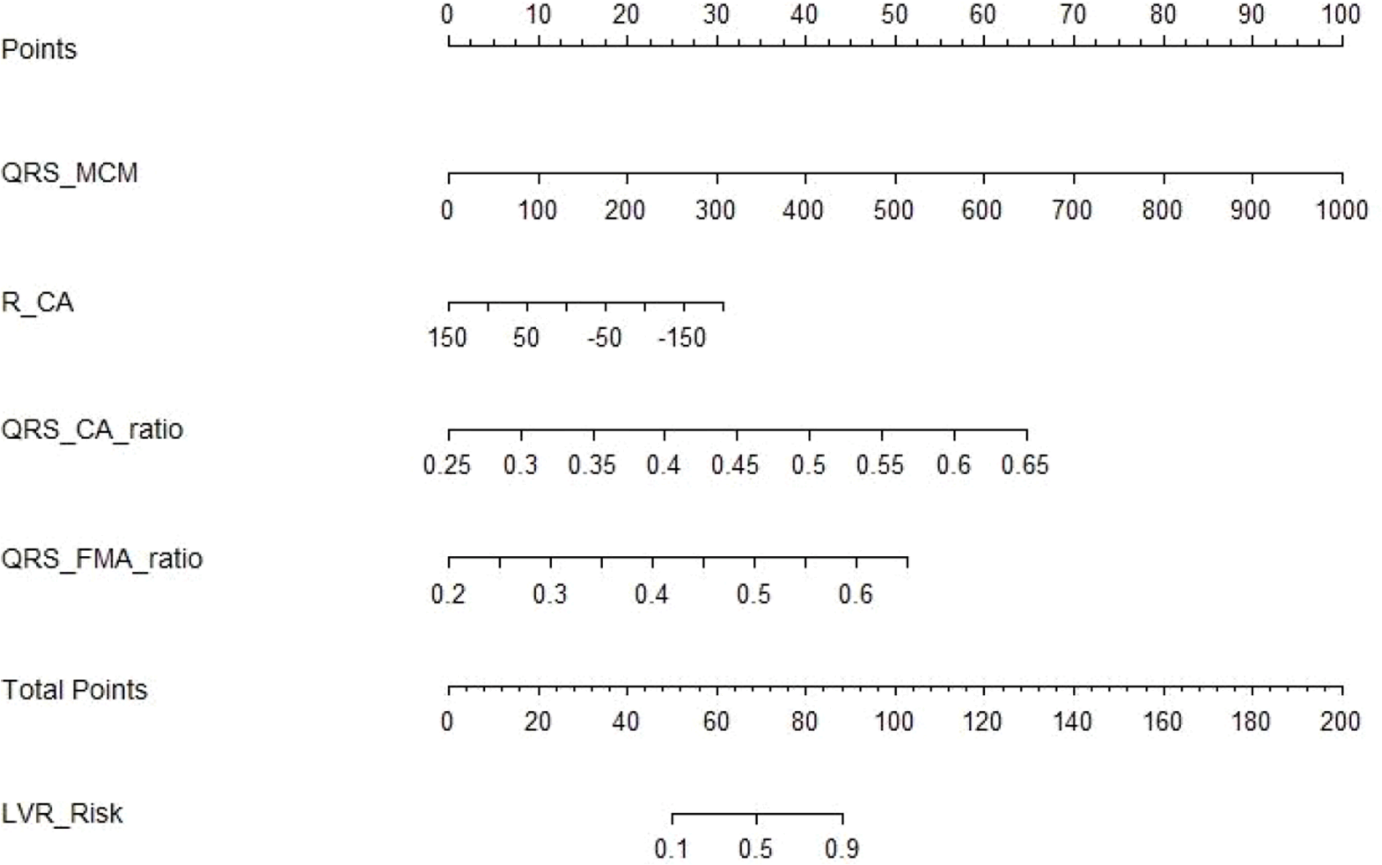
Nomogram of the four-parameter MCG associative model. The nomogram visually represents the contribution of each parameter (QRS_MCM, R_CA, QRS_CA ratio, QRS_FMA ratio) to the total points, which can be converted to an estimated probability of association with the ECHO-defined concentric structural phenotype. Note: This nomogram is a research illustration of the model structure and has not been validated for clinical use. The risk estimates shown are derived from the development set and may not generalize; this nomogram is for illustrative purposes only and is not intended for clinical decision-making.

A nomogram visualizing the contribution of each parameter in the model is provided in Figure 3. This is a research illustration of the model structure and has not been validated for clinical use.

### Subgroup analysis across Non-LVR, CR, and CH

A detailed comparison across the three phenotypic subgroups (Non-LVR, CR, CH) is provided in Supplementary Table S3. Consistent with the primary analysis, trends suggestive of a gradation in several MCG parameters were observed, though pairwise comparisons between CR and CH were not statistically significant after correction, likely due to limited sample size.

## Discussion

This comprehensive, cross-sectional study provides a detailed mapping of the relationships between a broad array of magnetocardiographic parameters and standard echocardiographic indices of left ventricular remodeling in patients with hypertension. By employing rigorous statistical controls for multiple comparisons and incorporating an internal validation step for a multivariate model, the present findings move beyond simple group differences to identify specific, statistically significant associations and a stable combined MCG signature linked to the structural remodeling phenotype. The central conclusions are that (1) The strength of the global depolarization current (QRS_MCM) and the relative timing of key electrical events within the cardiac cycle are correlated with the degree of concentric wall thickening and geometry; (2) Patients with ECHO-defined LVR exhibit a distinct MCG profile characterized by enhanced current moments, altered current vector orientation, and delayed relative depolarization timing; and (3) A parsimonious set of four MCG parameters demonstrates a stable associative pattern with the composite structural phenotype.

The positive correlations between QRS_MCM and wall thickness (IVSd, LVPWd) and particularly with RWT are mechanistically plausible. In concentric remodeling and hypertrophy, parallel replication of sarcomeres increases myocyte cross-sectional area and wall thickness. This anatomical change may result in a larger aggregate of electrically active tissue firing in a more synchronized manner during depolarization, thereby generating a stronger net current moment detectable by MCG.^23^ However, the observed correlations, while statistically significant, accounted for approximately 15% of the variance (e.g., QRS_MCM with RWT, *r^2^* = 0.15), indicating that most of the variability in MCG parameters is explained by factors not captured in this analysis, such as myocardial fibrosis, which can disrupt electrical synchronization; concurrent arrhythmias; and antihypertensive medications that may alter conduction properties.

The findings related to timing parameters, specifically the QRS_CA ratio and QRS_FMA ratio, are notable. The fact that these relative timing measures (as a proportion of the RR interval) showed robust correlations with structural indices, while their absolute time counterparts did not, suggests that in concentric remodeling, the temporal organization of depolarization relative to cycle length is altered. This may reflect altered ventricular conduction properties due to myocardial fibrosis, a known component of hypertensive heart disease, which can slow intramyocardial conduction velocity.^24^^,^^25^

### Comparison with standard ECG

A natural question is whether MCG offers any incremental value over standard ECG. In this cohort, conventional ECG voltage criteria (Sokolow-Lyon, Cornell voltage, Cornell product) showed only modest discrimination for the concentric structural phenotype (AUC 0.62–0.68), with low sensitivity (24%–36%). This is consistent with the well-known limitations of ECG criteria for detecting left ventricular hypertrophy, particularly in the setting of concentric remodeling without increased mass.^13^^,^^14^ The MCG model achieved a numerically higher AUC (0.856) in validation, but this comparison has important caveats. First, both analyses were performed in the same cohort, and the MCG model was developed to associate with the ECHO-defined outcome. Second, MCG requires specialized infrastructure, a magnetically shielded room, liquid helium, and substantial technical expertise, whereas ECG is inexpensive, portable, and universally available. Whether the observed difference in discrimination justifies the substantial additional cost and complexity cannot be answered by this exploratory study. Prospective studies directly comparing MCG and ECG against an independent reference standard (e.g., cardiac magnetic resonance) are needed to determine incremental value.

### Limitations and future directions

Several limitations must be acknowledged. First, the cross-sectional design establishes correlation but cannot determine causation or temporal sequence. Second, while internal validation mitigates overfitting, external validation in an independent cohort is essential. Third, the subgroup analysis comparing CR and CH was underpowered; these findings are exploratory and hypothesis-generating. Fourth, calibration of the multivariate model in the validation set could not be reliably assessed due to sample size, and the shift in sensitivity/specificity at a fixed threshold highlights that the model is not a clinically ready tool. Fifth, the authors did not perform a formal head-to-head comparison of ECG against an independent reference standard; the ECG analysis presented is descriptive and within the same cohort.

Future prospective, longitudinal studies are needed to test whether specific MCG parameters can identify “electrical remodeling” that predicts subsequent development of structural LVR or adverse events. Correlating MCG findings with tissue characterization from cardiac magnetic resonance (e.g., T1 mapping for diffuse fibrosis) could offer mechanistic insights. Additionally, direct comparison with ECG in a prospective design with a common reference standard is necessary to determine whether MCG offers incremental value.

## Conclusion

In this cross-sectional study, specific magnetocardiographic parameters, particularly those reflecting the global depolarization current strength (QRS_MCM) and the relative timing of electrical events (QRS_CA/FMA ratios), are significantly associated with echocardiographic indices of concentric left ventricular remodeling in patients with hypertension. A combination of MCG features shows a stable associative pattern with the concentric structural phenotype. These findings support a measurable link between MCG-based electrophysiological features and structural alterations. Further research is needed to determine whether MCG provides insights beyond those obtainable from simpler, more widely available tools such as the ECG, and to establish the temporal relationship between electrical and structural remodeling. Importantly, external validation in independent cohorts is required before any clinical application of these findings can be considered.

## Study perspective

Before Magnetocardiography (MCG) could be considered for clinical use in the assessment of hypertensive left ventricular remodeling, several critical steps are necessary. First, prospective longitudinal studies are needed to establish whether MCG parameters can predict the future development of structural remodeling or adverse cardiovascular events, thereby moving beyond cross-sectional associations. Second, direct head‑to‑head comparisons against an independent reference standard, preferably Cardiac Magnetic Resonance (CMR) imaging, are essential to determine whether MCG offers incremental diagnostic or prognostic value over standard tools such as the 12‑lead ECG. Third, cost‑effectiveness analyses should be performed, given that MCG currently requires specialized infrastructure, a magnetically shielded room, and liquid helium cooling, whereas ECG is inexpensive and universally available. Finally, external validation of any proposed MCG parameter combination in diverse, multicenter cohorts is mandatory to confirm the stability and generalizability of the observed associations. Until such evidence is available, MCG remains a promising research tool rather than a clinically established modality for evaluating left ventricular remodeling in hypertension.

## Ethical compliance statement

This study was approved by the *Ethics Committee of The Fourth Affiliated Hospital of Soochow University* (protocol number AP-JRB-SOP-011-03.0-02). All procedures performed in studies involving human participants were in accordance with the ethical standards of the institutional and/or national research committee and with the 1964 Helsinki declaration and its later amendments or comparable ethical standards.

## Informed consent statement

Written informed consent was obtained from all participants prior to study inclusion.

## Data availability statement

The datasets generated and analyzed during the current study are not publicly available due to patient privacy and confidentiality regulations of the institution, but are available from the corresponding author on reasonable request, subject to approval by the institutional ethics committee.

## Authors’ contributions

Junyi Zhang: Conceptualization; methodology; writing-original draft preparation; investigation.

Jianfeng Zhang: Data curation; software; formal analysis; visualization.

Yang Duan: Supervision; project administration; funding acquisition; writing, reviewing, and editing.

Yafeng Zhou: Conceptualization; resources; supervision; writing-reviewing and editing; validation.

## Fundings

This work was supported by grants from National Natural Science Foundation of China (81873486), the Science and Technology Development Program of Jiangsu Province-Clinical Frontier Technology (BE2022754), Clinical Medicine Expert Team (Class A) of Jinji Lake Health Talents Program of Suzhou Industrial Park (SZYQTD202102), Suzhou Key Discipline for Medicine (SZXK202129), Demonstration of Scientific and Technological Innovation Project (SKY2021002), Suzhou Dedicated Project on Diagnosis and Treatment Technology of Major Diseases (LCZX202132), Research on Collaborative Innovation of medical engineering combination (SZM2021014), Research on Collaborative Innovation of medical engineering combination (SZM2022003), Suzhou Key Laboratory of Diagnosis and Treatment of Panvascular Diseases (SZS2023021), Gusu Talent Program (GSWS2022119). The funders had no roles in study design, data collection and analysis, decision to publish, or preparation of the manuscript.

## Declaration of competing interest

The authors declare no conflicts of interest.

## Appendix Supplementary materials

Image, application 1

Image, application 2

Image, application 3

## Footnotes

Supplementary material associated with this article can be found, in the online version, at doi:10.1016/j.clinsp.2026.101044.
