# Supplementary material for: Exploring the association between magnetocardiographic parameters and echocardiographic indices of concentric left ventricular remodeling in hypertension: a cross-sectional correlation study
Source: Clinics (Sao Paulo). 2026 Jul 29;81:101044. doi: 10.1016/j.clinsp.2026.101044 (PMC13451904; doi:10.1016/j.clinsp.2026.101044)
Supplement: Supplementary file 1 [file mmc2.pdf]

**CRediT authorship contribution statement**

**Junyi Zhang:** Conceptualization, Methodology, Writing – Original draft preparation, Investigation. **Jianfeng Zhang:** Data curation, Software, Formal analysis, Visualization. **Yang Duan:** Supervision, Project administration, Funding acquisition, Writing – Reviewing and Editing. **Yafeng Zhou:** Conceptualization, Resources, Supervision, Writing – Reviewing and Editing, Validation.
