# Supplementary material for: Exploring the association between magnetocardiographic parameters and echocardiographic indices of concentric left ventricular remodeling in hypertension: a cross-sectional correlation study
Source: Clinics (Sao Paulo). 2026 Jul 29;81:101044. doi: 10.1016/j.clinsp.2026.101044 (PMC13451904; doi:10.1016/j.clinsp.2026.101044)
Supplement: Supplementary file 2 [file mmc3.pdf]

Ethical Compliance Statement

This study was approved by ***Ethics Committee of The Fourth Affiliated Hospital of Soochow University (protocol number AP-JRB-SOP-011-03.0-02).*** All procedures performed in studies involving human participants were in accordance with the ethical standards of the institutional and/or national research committee and with the 1964 Helsinki declaration and its later amendments or comparable ethical standards.
