# Supplementary material for: Exploring the association between magnetocardiographic parameters and echocardiographic indices of concentric left ventricular remodeling in hypertension: a cross-sectional correlation study
Source: Clinics (Sao Paulo). 2026 Jul 29;81:101044. doi: 10.1016/j.clinsp.2026.101044 (PMC13451904; doi:10.1016/j.clinsp.2026.101044)
Supplement: Supplementary file 3 [file mmc1.docx]

CLINICS-D-25-01473_Supplementary material

**Table S1** Diagnostic performance of conventional ECG voltage criteria for detecting echocardiography defined left ventricular remodeling in the overall cohort (n = 220).

| **ECG criterion** | **AUC (95% CI)** | **Sensitivity (%)** | **Specificity (%)** | **Youden index** |
| --- | --- | --- | --- | --- |
| Sokolow‑Lyon voltage (SV₁ + RV₅/V₆) ≥ 35 mm | 0.62 (0.55–0.69) | 24.3 | 89.5 | 0.138 |
| Cornell voltage (RaVL + SV₃) | 0.66 (0.59–0.73) | 33.9 | 86.7 | 0.206 |
| Cornell product (Cornell voltage × QRS duration) ≥ 2440 mm·ms | 0.68 (0.61–0.75) | 36.5 | 85.7 | 0.222 |

AUC, Area Under the receiver operating Characteristic curve; CI, Confidence Interval.

**Table S2** Sensitivity analysis using Benjamini-Hochberg False Discovery Rate (FDR) correction for correlations between MCG and ECHO parameters.

| **MCG Parameter** | **ECHO Parameter** | **Correlation Coefficient (r)** | **p-value** | **FDR q-value** |
| --- | --- | --- | --- | --- |
| QRS_MCM | IVSd | 0.388 | <0.0001 | <0.0001 |
| QRS_MCM | LVPWd | 0.333 | <0.0001 | <0.0001 |
| QRS_MCM | RWT | 0.392 | <0.0001 | <0.0001 |
| QRS_CA ratio | IVSd | 0.328 | <0.0001 | <0.0001 |
| QRS_CA ratio | RWT | 0.339 | <0.0001 | <0.0001 |
| QRS_FMA ratio | IVSd | 0.299 | <0.0001 | <0.0001 |
| QRS_FMA ratio | RWT | 0.327 | <0.0001 | <0.0001 |
| R_PD | LVMI | 0.244 | 0.0002 | 0.0005 |
| QRS_MCM | LVMI | 0.197 | 0.003 | 0.005 |
| R_CA | LVMI | -0.186 | 0.006 | 0.008 |
| QRS_Intergral | RWT | 0.182 | 0.007 | 0.009 |
| R_CA ratio | IVSd | 0.176 | 0.009 | 0.011 |

Note: Only correlations with FDR q-value < 0.05 are shown. All correlations are Spearman’s rho.

**Table S3** Comparison of MCG parameters among Non-LVR, Concentric Remodeling (CR), and Concentric Hypertrophy (CH) groups.

| **Parameter** | **Non‒LVR**  **(n = 105)** | **CR**  **(n = 65)** | **CH**  **(n = 50)** | **p-value (Overall)** | **p-value (CR vs. Non-LVR)** | **p-value (CH vs. Non-LVR)** | **p-value (CH vs. CR)** | **Cohen’s d (CH vs. CR) [95% CI]** |
| --- | --- | --- | --- | --- | --- | --- | --- | --- |
| QRS_Integral | 2801.2 ± 892.3 (2628.5‒2973.9) | 3205.6 ± 1056.7 (2948.6‒3462.6) | 3350.8 ± 1123.4 (3033.6‒3668.0) | <0.001 | 0.012 | <0.001 | 0.465 | 0.13 [-0.19, 0.45] |
| QRS_MCM | 185.4 ± 45.2 (176.7‒194.1) | 225.6 ± 52.1 (212.7‒238.5) | 240.3 ± 58.9 (223.6‒257.0) | <0.001 | <0.001 | <0.001 | 0.145 | 0.27 [-0.05, 0.59] |
| R_MCM | 160.8 ± 38.7 (153.3‒168.3) | 195.2 ± 44.5 (184.1‒206.3) | 210.1 ± 50.2 (196.0‒224.2) | <0.001 | <0.001 | <0.001 | 0.098 | 0.31 [-0.01, 0.63] |
| R_CA | 44.2 ± 8.9 (42.5‒45.9) | 40.1 ± 9.3 (37.8‒42.4) | 38.8 ± 8.5 (36.4‒41.2) | <0.001 | 0.005 | <0.001 | 0.480 | -0.14 [-0.46, 0.18] |
| QRS_CA ratio | 0.32 ± 0.06 (0.31‒0.33) | 0.38 ± 0.07 (0.36‒0.40) | 0.40 ± 0.08 (0.38‒0.42) | <0.001 | <0.001 | <0.001 | 0.157 | 0.27 [-0.05, 0.59] |
| QRS_FMA ratio | 0.34 ± 0.07 (0.33‒0.35) | 0.41 ± 0.08 (0.39‒0.43) | 0.43 ± 0.09 (0.40‒0.46) | <0.001 | <0.001 | <0.001 | 0.210 | 0.23 [-0.09, 0.55] |

Data presented as mean ± SD; p-values adjusted for multiple comparisons using Bonferroni correction.
